# On Zagreb coindices and Mostar index of $TiO_2$ nanotubes

**DOI:** 10.1038/s41598-023-40089-6

**Published:** 2023-08-22

**Authors:** Muhammad Imran, Mehar Ali Malik, Muhammad Aqib, Gul I Hina Aslam, Amjad Ali

**Affiliations:** 1 Department of Mathematical Sciences, United Arab Emirates University, P.O. Box 15551 Al Ain, United Arab Emirates; 2 Department of Mathematics, Riphah International University, 54000 Lahore, Pakistan; 3 Department of Mathematics, College of Electrical & Mechanical Engineering (CEME), National University of Sciences and Technology, 44000 Islamabad, Pakistan; 4 Pakistan Navy Engineering College, National University of Sciences and Technology, Karachi, Pakistan; 5 Faculty of Science and Technology, University of Debrecen, Debrecen, 4001 Hungary

**Keywords:** Biochemistry, Chemical biology, Chemistry, Materials science, Nanoscience and technology

## Abstract

Topological indices are valuable tools in predicting properties of chemical compounds. This study focuses on degree-based topological indices, which have shown strong correlations with various physico-chemical properties such as boiling points and strain energy. Specifically, we applied these indices to titania nanotubes $TiO_2$ and explored the vertex and edge versions of the Mostar index. These findings provide insights into the properties of $TiO_2$ nanotubes and contribute to the development of topological indices for predicting the behavior of other chemical compounds.

## Introduction

With the aid of mathematical tools, mathematical chemistry discusses and predicts the chemical properties of molecules by using its structure. It is a branch of mathematics combining graph theory with mathematical chemistry to model chemical phenomena mathematically. Chemical sciences rely heavily on this theory.

Molecular graphs are simple graphs where atoms are represented by vertices, and chemical bonds are represented by edges. It is common for molecular graphs to omit hydrogen atoms. Consider a molecular graph *G* where its vertex set is $V(G)={v_1,v_2,\ldots ,v_n}$ and its edge set is *E*(*G*). The order of graph *G* is represented by $|V(G)| = n$. An edge in *E*(*G*) with end vertices *u* and *v* is denoted by *uv*. The degree of a vertex is the number of edges incident on *v* and denoted as $d_G(v)$ or $d_v$. That is, $d_v=|N(v)|$. The neighborhood $N_G(v)$ of a vertex $v\in V(G)$ is the set of vertices adjacent to *v*, that is, $N_G(v) = \{w\in V(G) ~|~ vw \in E(G)\}$. The sum of degrees of all the vertices adjacent to a vertex $u\in V(G)$ is defined as $S_u = \sum \nolimits _{v\in N(u)} d_v$.

A set $S\subseteq E(G)$ is called an edge-cut of a connected graph *G* if $G-S$ is disconnected, where $G-S$ is the graph obtained from *G* by deleting all the edges of *S* from *G*. The distance between two vertices is defined as the number of edges in shortest path connecting them. It is also called graph geodesic distance. Let an edge $e=uv$ and *v* be a vertex then the distance between edge *e* and vertex *v* is defined as $d(e, v) = \min \{ d(x, v),d(y, v) \}$.

Two edges $e = uv$ and $f = xy$ of a graph G are called codistant, denoted as *ecof*, if $d(v,x) = d(v,y) + 1 = d(u,x) + 1 = d(u,y)$. The relation *co* is reflexive and symmetric but not necessarily transitive. When relation *co* is transitive in a graph *G*, then *G* is called a co-graph. In this case, *E*(*G*) is union of disjoint equivalence classes of the relation *co* known as orthogonal cuts.

For $e = uv \in E(G)$, the number of vertices and edges lying closer to an end-vertex of *e* are define as $N_u(e)$ and $M_u(e)$. The $n_u(e)$ define as the number of vertices of *G* closer to *u* than *v*. Similarly, the $m_u (e)$ define as the number of edges of *G* closer to *u* than *v*. These number are obtained from the sets defined as follows.

1 $$\begin{aligned} N_u (e\,|\,G)= & {} \{x \in V(G) ~|~ d_G (u,x) < d_G (v,x)\}, \end{aligned}$$

2 $$\begin{aligned} M_u (e\,|\,G)= & {} \{f \in E(G) ~|~ d_G (u, f ) < d_G (v,f )\}. \end{aligned}$$

## Topological indices

A topological index is a molecular graph invariant that correlates the physico-chemical properties of a molecular graph with a number. The first topological index was introduced in 1947 to calculate the boiling points of paraffins. This numerical representation of a molecular graph is useful in the quantitative structure-property relationship (QSPR), in communication, cryptography, and facility location, as well as other applications.

This index was originally defined for trees to correlate the certain physico-chemical properties of alkanes, alcohols, amines and their compounds. Hosoya^1^ defined the notion of Wiener index for any graph *G* as

3 $$\begin{aligned} W(G) = \sum _{\{u,v\}\subseteq V(G)}d(u,v). \end{aligned}$$

Gutman and Trinajstic^2,3^ investigated the dependence of total $\pi$-electron energy of an alternate hydrocarbon, where they encountered the terms denoted by $M_1$ and $M_2$ commonly known as Zagreb indices of first and second kind and defined as follows.

4 $$\begin{aligned} M_1(G)= & {} \sum _{u\in V(G)} d^2(u), \end{aligned}$$

5 $$\begin{aligned} M_2(G)= & {} \sum _{uv\in E(G)} d(u)d(v). \end{aligned}$$

Furtula and Gutman^4^ introduced the forgotten topological index as:

6 $$\begin{aligned} F(G) = \sum _{u\in V(G)} d^3(u) = \sum _{uv\in E(G)} (d^2(u)+d^2(v)). \end{aligned}$$

In this paper^4^, Forgotten topological index is also denoted as F-Index.

In the similar way, De et al.^5^ defined the F-coindex as follows.

7 $$\begin{aligned} {\overline{F}}(G) = \sum _{u\notin V(G)} d^3(u) = \sum _{uv\notin E(G)} (d^2(u)+d^2(v)). \end{aligned}$$

Doslic^6^ defined the first and second Zagreb coindices as follows.

8 $$\begin{aligned} {\overline{M}}_1 (G)= & {} \sum _{uv \notin E(G)} [d(u) + d(v)], \end{aligned}$$

9 $$\begin{aligned} {\overline{M}}_2 (G)= & {} \sum _{uv \notin E(G)} d(u)d(v). \end{aligned}$$

The Mostar index was defined by Doslić et al.^7^ as follows.

10 $$\begin{aligned} Mo(G) = \sum _{e=uv \in E(G)} |n_u(e)-n_v(e)|. \end{aligned}$$

The edge Mostar index was defined by Havare et al.^8^ as follows.

11 $$\begin{aligned} Mo_e(G) = \sum _{e=uv \in E(G)} |m_u(e)-m_v(e)|. \end{aligned}$$

## Motivation

Niko^9^ studied the Mostar index of weighted graphs and applied their results to benzenoid systems. Shehaz et al.^10^ studied the Mostar index of several graph operations including lexicographic, Cartesian, corona product and more. Doslic et al.^11^ studied the extremal values of the Mostar index and obtained extremal trees. They also applied the results to some large classes of chemically interesting graphs. Akbar and Doslic^12^ presented various modifications and studied bounds and extremal results related to Mostar index. Ghorbani et al.^13^ studied the vertex-orbits with respect to the Mostar index under the action of automorphism group. They also studied the graphs with respect to value of the Mostar index equal to one.

The Zagreb coindices were recently studied in^14–19^ and details on the relations between Zagreb indices and coindices can be found in^20,21^. Two distance-based indices of some graph operations were studied in Ref.^22^.

The atom-bond conenctivity index and geometric-arithmetic index of some fullerenes was studied in Ref.^23^. Ghorbani et al.^24^ studied the nullity of an infinite class of nanostar dendrimers.

In recent years, these indices have gained significant attention due to their potential applications in drug discovery, material science, and network analysis. Havare^8,25^ studied the Mostar index of bridge graphs and showed its relevance in modeling the electronic properties of nanotubes. Similarly, the study by Kier and Hall demonstrated the usefulness of the Zagreb and Forgotten indices in predicting the toxicity and bioactivity of chemical compounds. These indices contribute to the understanding of the physico-chemical properties of $TiO_2$ nanotubes.

Topological index provide a bridge that transform the molecule graph into a number. By using different topological indices, we can exercised for designing biological, physico-chemical, toxicological, pharmacologic and other characteristics of chemical compounds. Ashrafi et al.^26^ studied the infinite classes of siloxane and POPAM dendrimers and derive their Zagreb eccentricity indices, eccentric-connectivity and total-eccentricity indices.

## Methodology

We use the information of degrees of vertices in molecular graphs of titania nanotubes and the complement of these graphs to obtain some degree-based indices and coindices of titania nanotubes in “[Sec Sec7]”. In “Mostar index of titania nanotubes”, we use the edge-cuts defined in Refs.^27,28^ and use the $n_u,n_v$ partition of the edge set of titania nanotubes to obtain the Mostar index that is a recently defined distance-based topological index. Then in the last section, we represent these results graphically and give a comparative analysis of the obtained results.

## Titania nanotubes $TiO_2$

As a well-known semiconductor with a numerous technological applications, titania is comprehensively studied in materials science. Titania nanotubes were systematically synthesized during the last 10–15 years using different methods and carefully studied as prospective technological materials. Since the growth mechanism for $TiO_2$ nanotubes is still not well defined, their comprehensive theoretical studies attract enhanced attention. The $TiO_2$ sheets with a thickness of a few atomic layers were found to be remarkably stable^29^.

**Figure 1:**
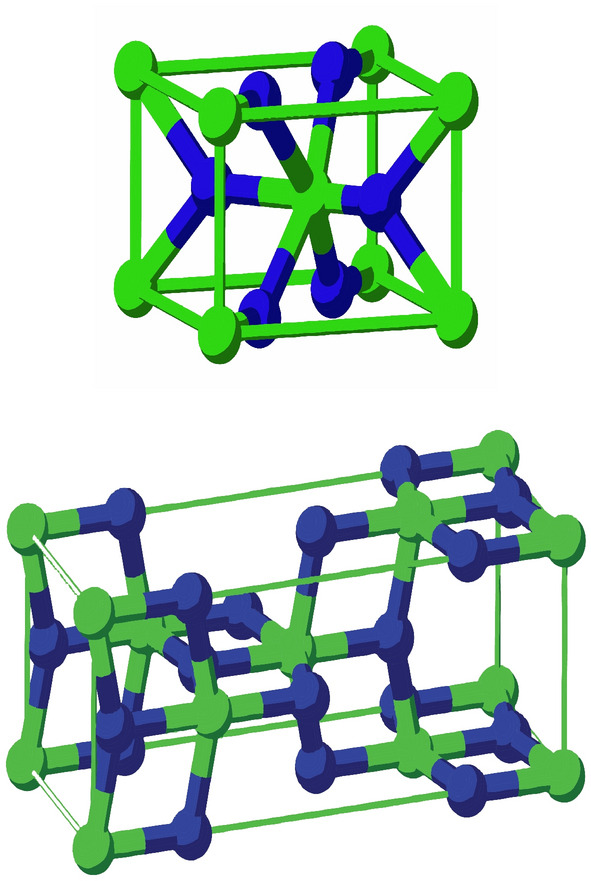
Molecular structure of $TiO_2$-nanotubes for $n=1,2,m=1,2$, image source^30^.

**Figure 2:**
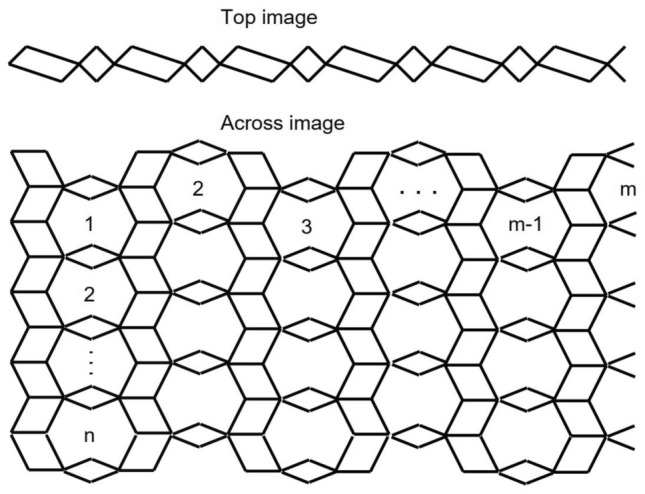
The graph of $TiO_2[m,n]$-nanotubes, for $m=6$ and $n=4$. Image created by Mayura^31^.

**Figure 3:**
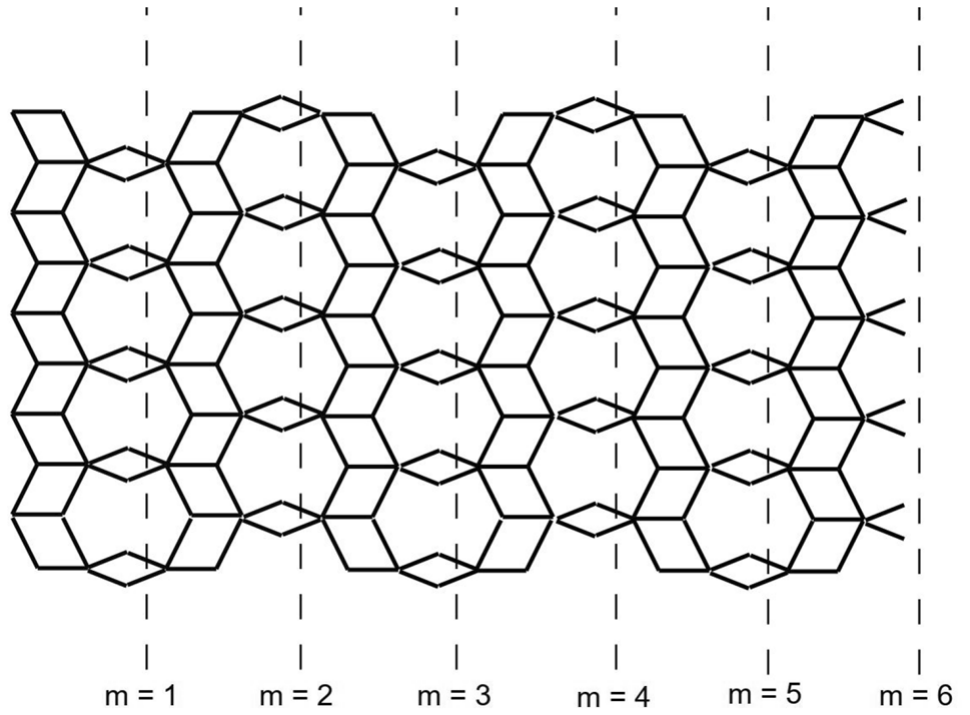
The graph of $TiO_2[m,n]$-nanotubes, for $m=6$ and $n=4$. Image created by Mayura^31^.

The graph of the Titania nanotubes $TiO_2[m,n]$ is presented in Fig. 1 where *m* denotes the number of octagons in a row and *n* denotes the number of octagons in a column of the titania nanotube in Figs. 2 and 3.

The next section deals with computation of first and fourth versions of atom-bond connectivity index and first and fifth versions of geometric-arithmetic index of Titanium nanotubes $TiO_2$.

## Applications of titania nanotubes

**Figure 4:**
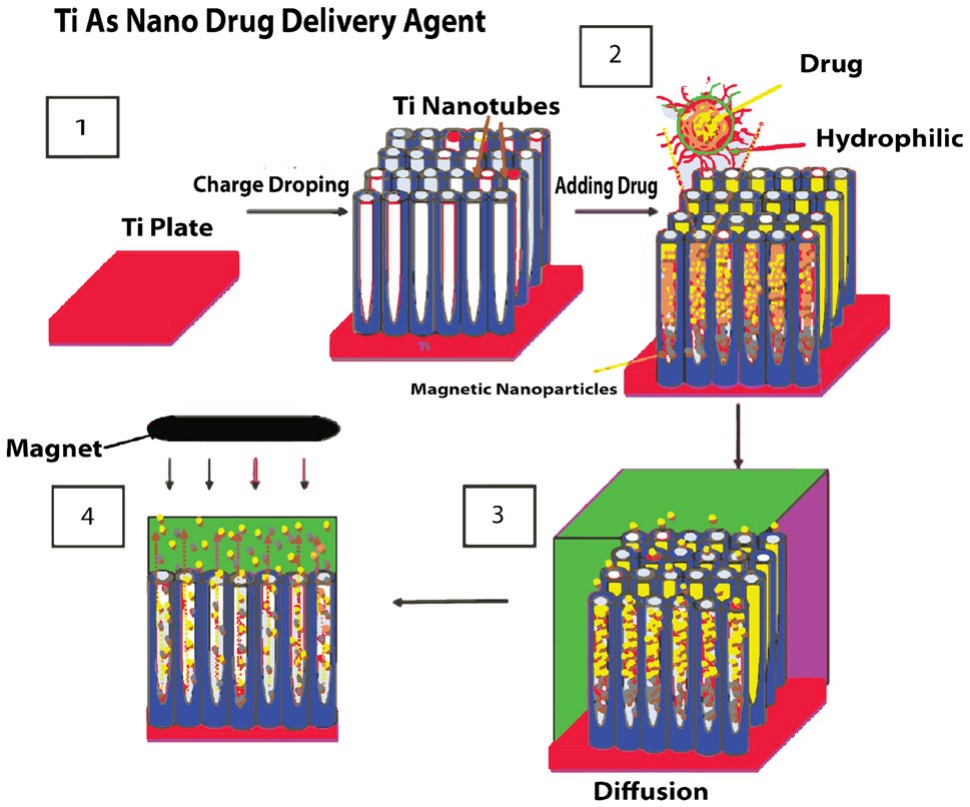
$TiO_2$ working as drug delivery agent in industry. Image created in Adobe^33^.

Titanium dioxide $(TiO_2)$ nanotubes is that compound that contain plentiful variation with different compounds that have emerged in various fields of technology such as medicine, energy and biosensing. Sevda et al.^32^ studied that $(TiO_2)$ nanotubes can react with different diversified drugs like antibiotics, osteoporosis drugs and anticancer. As shown in Fig. 4, the titanium tubes react with basic chemical molecule and made polymer with it and worked as drug delivery agent. Furthermore, $TiO_2$ nanotubes and their derivatives are very helpful in to overcome human pathogenic microorganisms. Moreover, $TiO_2$ have wide uses and applications in medical implants, antibacterial fields, drug delivery and nano biosensing.

Irshad et al.^34^ studied the application of $TiO_2$ nano particles (*NPs*) with different characteristics and their wide range of applications. The $TiO_2$ NPs are mainly used for the cleanses of polluted water and positively affected the plant physiology especially under abiotic stresses but the response varied with types, size, shapes, doses, duration of exposure, metal species along with other factors.

**Figure 5:**
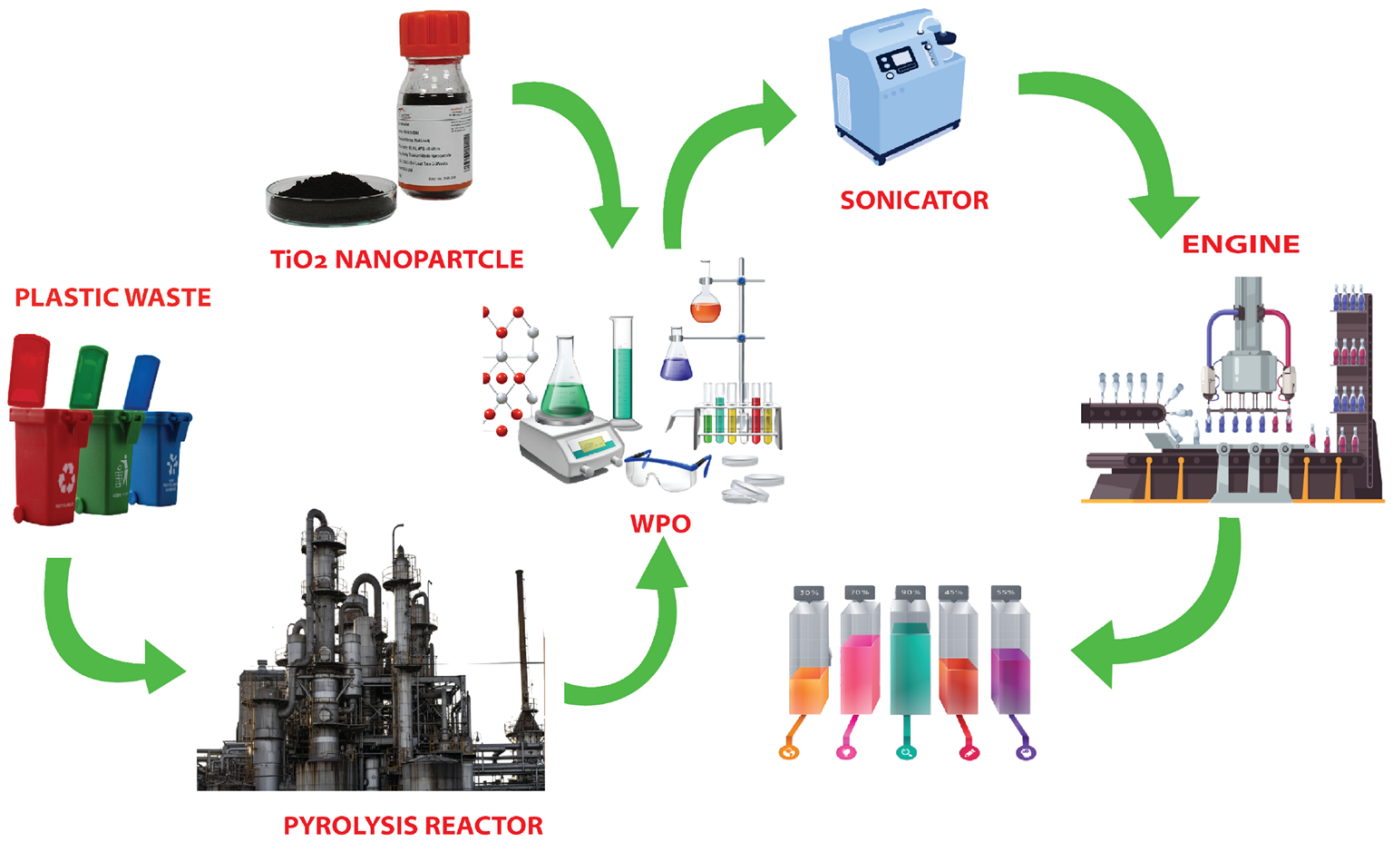
The $TiO_2$ nanotubes as disposal agent of plastic polymer. Image created in Adobe^33^.

Now a days, plastic is the man-made pollution which become the largest problem of the world due to its ubiquitous presence and unknown threat to living organisms. Nabi et al. studied in^35^ $TiO_2$ based photocatalysis has been typically highlighted as a degradation method for plastics treatment. As discussed in Fig. 5, first plastic garbage is proceed through Pyrolysis process and then $TiO_2$ nano-particles are added in the mixture. After that, this mixture pass through the sonic mechine to separate in different types of basic molecules that can be reused. Degradation performance of $TiO_2$ can be enhance by coupling with carbon, nitrogen and vitamin C to some extent for specific plastics decomposition. $TiO_2$ based photocatalytic system should efficiently decompose from single to multiple types of polymers.

## Some degree-based indices and coindices of $TiO_2$ nanotubes

First, we obtain the edge partition of the graph of these nanotubes with respect to the degrees of end-vertices of all edges in $E(TiO_2)$. This partition will help us in applying the formulas of the mentioned indices,

With each edge *uv*, we associate two pairs $(d_u,d_v)$. The edge partition of Titania nanotubes $TiO_2$ with respect to the degrees of the end-vertices of edges is given by Table 1.

**Table 1:** The $(d_u,d_v)$-type edge partition of Titania nanotubes.

$(d_u,d_v)$	No. of edges
(2, 4)	6*n*
(2, 5)	$$2n+4mn$$
(3, 4)	2*n*
(3, 5)	$$6n(m-1)+4n$$

### Theorem 7.1

*Let* *G* *denote the graph of 2D-lattice of* $TiO_{2}$ *nanotubes*. *Then the F-index of* *G* *is given by*,

$$\begin{aligned} F(G) = {\left\{ \begin{array}{ll} 320n-128, &{} \text {when }m=1\\ 84+640mn-212m-320n, &{} \text {when }m>1. \end{array}\right. } \end{aligned}$$

### Proof

The edge partition of *G* based on the degrees of end-vertices are given in Table 2 along with their frequencies.

**Table 2:** The frequencies of each type of edge *uv* in two dimensional lattice of titania nanotubes *T*[*m*, *n*], for $m=1$ and $m>1$.

$f(d_u,d_v)$	$m=1$	$m>1$
*f*(2.2)	4	2
*f*(2, 3)	0	4
*f*(2, 4)	$8+16(n-1)$	$6+12(n-1)$
*f*(2, 5)	0	$8mn-4(m+n)+2$
*f*(3, 3)	0	$4+6(m-2)$
*f*(3, 4)	0	$2+4(n-1)$
*f*(3, 5)	0	$12mn-6m+8(1-2n)$

Now, by definition, the F-index of *G* is given by,

$$\begin{aligned} F(G)= \sum _{uv\in E(G)}(d_u^2+d_v^2)f(u,v). \end{aligned}$$

We compute the result in two cases. When $m=1$, we have

$$\begin{aligned} F(G)&= 4(2^2+2^2)+0+(2^2+4^2)(8+16(n-1))\\&= 320n-128. \end{aligned}$$

When $m>1$, we use Table 2 to obtain the following result.

$$\begin{aligned} F(G)&= (2^2+2^2)(2^2+3^2)(4)+(2^2+4^2)(6+12(n-1)\\&\quad +(2^2+5^2)(8mn-4(m+n)+2)\\&\quad +(3^2+3^2)(4+6(m-2))+(3^2+4^2)(2+4(n-1))\\&\quad +(3^2+5^2)(12mn-6m+8(1-2n))\\&= 84+640mn-212m-320n. \end{aligned}$$

$$\square$$

Now we calculate the F-coindex and first Zagreb coindex of titania nanotubes $TiO_2$. The coindices of graphs are defined in terms of the edges of the complement of a graph. We use the definition of the complement of a graph and represent the coindex in a simpler way. Obviously, $E(G) \cup E({\overline{G}}) = E(K_n)$, where $K_n$, represents a complete graph. So if *v* has degree $d_v$ in *G* then degree of the same vertex will be $n - 1 - d_v$, in ${\overline{G}}$. For each $u\in V(G)$, let us denote $N_u = n - 1 - d_u$. Then

$$\begin{aligned} {\overline{F}}(G)=\sum _{u\in G}d_u^2\times N_u. \end{aligned}$$

**Figure 6:**
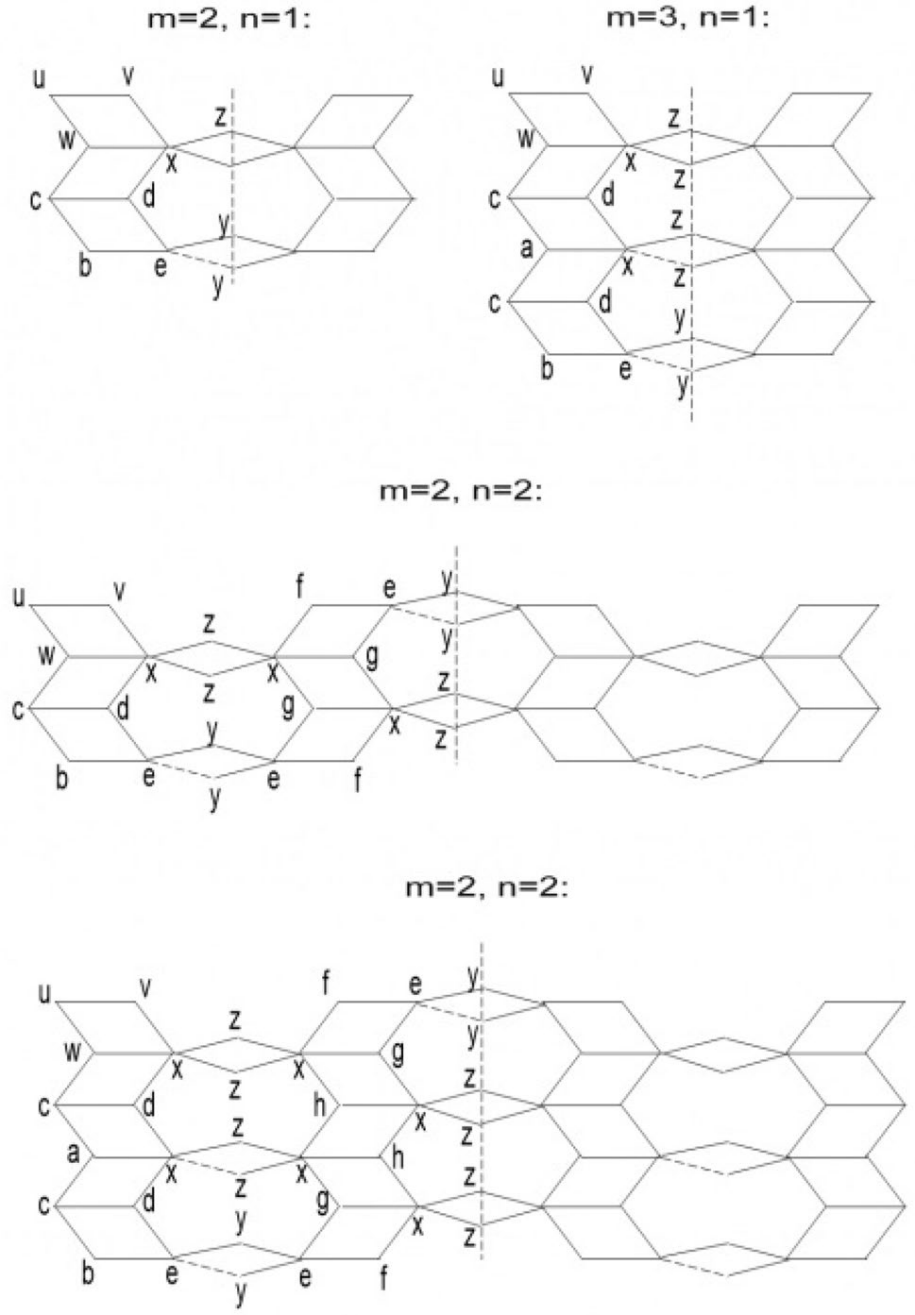
The lattices of $TiO_{2}[m,n]$ for $m=2$ and $n=1,2$.

**Figure 7:**
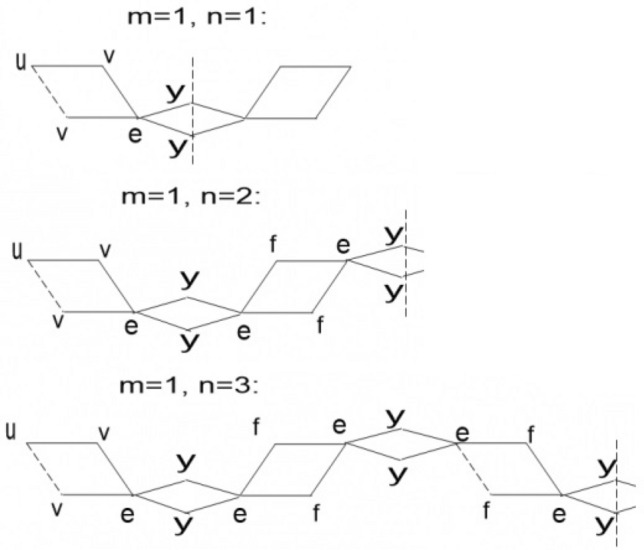
The lattices of $TiO_{2}[m,n]$ for $m=1$ and $n=1,2,3$.

Now, we proceed towards our main calculation. First we present some graphs of $TiO_2$ nanotubes that describe the main edge classes with respect to the degrees of end-vertices of all edges. Three graphs of titania nanotubes $TiO_2[3,1]$, $TiO_2[2,1]$ and $TiO_2[2,2]$ are presented in Fig. 6, where edges of different types are highlighted with different alphabets. Figure 7 gives different dimensional 2D lattices of $TiO_2$ nanotubes with $m=1$. Then we present tables summarizing the information about these edge classes and non-adjacencies of all the vertices, see Tables 3 and 4. These tables are used to obtained the next results.

**Table 3:** Degrees, frequencies and non-adjacencies of the representative vertices in the 2D lattice of $TiO_2[m,n]$, for $m=1$.

Representative	Degree	Frequency	Non-adjacency ($N_u$)
*u*	2	1(2)=2	$12n-5$
*v*	2	2(2)=4	$12n-5$
*y*	2	$2+4(n-1)$	$12n-5$
*e*	4	$2+4(n-1)$	$12n-7$
*f*	2	$0+4(n-1)$	$12n-5$

**Table 4:** Degrees, frequencies and non-adjacencies of the representative vertices in the 2D lattice of $TiO_2[m,n]$, for $m>1$.

Representative	Degree	Frequency	Non-adjacency ($N_u$)
*u*	2	2	$12mn-2m-3$
*v*	2	2	$12mn-2m-3$
*w*	3	2	$12mn-2m-4$
*x*	5	$4mn-2m-4n+2$	$12mn-2m-6$
*y*	2	$4n-2$	$12mn-2m-3$
*z*	2	$4mn-2m-4n+2$	$12mn-2m-3$
*a*	3	$2m-4$	$12mn-2m-4$
*b*	2	2	$12mn-2m-3$
*c*	3	$2m-2$	$12mn-2m-4$
*d*	3	$2m-2$	$12mn-2m-4$
*e*	4	$4n-2$	$12mn-2m-5$
*f*	2	$4n-4$	$12mn-2m-3$
*g*	3	$4n-4$	$12mn-2m-4$
*h*	3	$4mn-8n$	$12mn-2m-4$

### Theorem 7.2

*Let* *G* *denote the graph of 2D-lattice of* $TiO_{2}$ *nanotubes*. *Then the F-coindex of* *G* *is given as follows*:

$$\begin{aligned} {\overline{F}}(G) = {\left\{ \begin{array}{ll} 1152n^2-992n+224; &{} m=1 \\ 1824m^2n^2-352m^2n-672mn^2+8m^2 &{} \\ -1352mn+220m+376n+172; &{} m>1 \end{array}\right. } \end{aligned}$$

### Proof

From the figures shown, it is evident that there are 14 distinct vertex representations, based on degree, non-adjacency and frequency.

Now, the F-coindex of *G* is given by

$$\begin{aligned} {\overline{F}}(G)= \sum _{u\in T_2(m,n)} d_{u}^{2}\cdot (N_u)\cdot (frequency). \end{aligned}$$

We compute the result in the following two cases.

When $m=1$, using Table 3 we get;

$$\begin{aligned} {\overline{F}}(G)= & {} 2^2(12n-5)(2)+2^2(12n-5)(4)+2^2(12n -5)(4n-2)\\{} & {} +4^2(12n-7)(4n-2)+2^2(12n-5)(4(n-1)) \\= & {} 1152n^2-992n+256. \end{aligned}$$

When $m>1$, Using Table 4 we get the F-coindex of the graph *G* as follows.

$$\begin{aligned} {\overline{F}}(G)= & {} 2^2(12mn-2m-3)(2)+2^2(12mn-2m-3)(2)\\{} & {} +3^3(12mn-2m-4)(2)+5^5(12mn-2m-6)\\{} & {} (4mn-2m-4n+2)+2^2(12mn-2m-3)(4n-2)\\{} & {} +2^2(12mn-2m-3)(4mn-2m-4n+2)+3^3(12mn-2m-4)\\{} & {} (2m-4)+2^2(12mn-2m-3)(2)+3^3(12mn-2m-4)(2m-2)\\{} & {} +3^2(12mn-2m-4)(2m-2)+4^2(12mn-2m-5)(4n-2) \\{} & {} +2^2(12mn-2m-3)(4n-4)+3^3(12mn-2m4)(4n-4)\\{} & {} +3^2(12mn-2m-4)(4mn-8n)\\= & {} 1824m^2n^2-352m^2n-672mn^2+8m^2-1352mn+220m\\{} & {} +376n+172. \end{aligned}$$

$$\square$$

### Theorem 7.3

*Let* *G* *denote the graph of 2D-lattice of* $TiO_{2}$ *nanotubes*. *The first Zagreb coindex of* *G* *is given as follows*:

$$\begin{aligned} {\overline{M}}_1(G) = {\left\{ \begin{array}{ll} 288n^2 - 184n + 28; &{} m=1 \\ 480m^2n^2-32m^2n-96mn^2-8m^2 &{} \\ -464mn+48m+64n+88; &{} m>1 \end{array}\right. } \end{aligned}$$

### Proof

From the figures shown, it is evident that there are 14 distinct vertex representations, based on degree, non-adjacency and frequency. Now, the F-coindex of *G* is given by

$$\begin{aligned} {\overline{M}}_1(G)= \sum _{u\in V(G)}d_{u} \cdot (N_u)\cdot (frequency). \end{aligned}$$

When $m=1$, we use Table 3 to obtain the following.

$$\begin{aligned} {\overline{M}}_1(G)&=2(12n-5)(2)+2(12n-5)(4)+2(12n-5)(4n\\&\quad -2)+4(12n-7)(4n-2) + 2(12n-5)(4(n-1)) \\&= 4(12n-5)+8(12n-5)+(8n-4)(12n-5)\\&\quad +(8n-4)(12n-7)+(8n-8)(12n-5) \\&= 288n^2 - 184n + 28. \end{aligned}$$

Similarly, when $m>1$, we use Table 4 to obtain the first Zagreb coindex as follows.

$$\begin{aligned} {\overline{M}}_1(G)&= 2(12mn-2m-3)(2)+2(12mn-2m\\&\quad -3)(2)+3(12mn-2m-4)(2)+5(12mn\\&\quad -2m-6)(4mn-2m-4n+2)+2(12mn\\&\quad -2m-3)(4n-2)+2(12mn-2m-3)(4mn\\&\quad -2m-4n+2)+3(12mn-2m-4)(2m-4)\\&\quad +2(12mn-2m-3)(2)+3(12mn-2m-4)(2m\\&\quad -2)+3(12mn-2m-4)(2m-2)+4(12mn\\&\quad -2m-5)(4n-2)+2(12mn-2m-3)(4n-4)\\&\quad +3(12mn-2m-4)(4n-4)+3(12mn-2m\\&\quad -4)(4mn-8n)\\&= 480m^2n^2-32m^2n-96mn^2-8m^2\\&\quad -464mn+48m+64n+88. \end{aligned}$$

This completes the proof. $\square$

## Mostar index of titania nanotubes

In this section, we calculate the Mostar index if the molecular graphs of 2-dimensional titania nanotubes. So, first we have to obtain the all types of edge-cuts of $TiO_2$ nanotubes that are discussed by Imran et al.^27^.

**Table 5:** All types of edge-cuts with their cardinalities.

Case	Cut-type	Number of cuts	Size of cuts
When $m>n$	$A_i$	$1\le i\le n$	4*i*
	$B_i$	$1\le i\le m-n-1$	4*n*
	$C_i$	$1\le i\le n$	$2(2i-1)$
	$Y_i$	$1\le i\le n$	2*m*
	$Z_i$	$1\le i\le 2n-1$	2*m*
When $m\le n$	$A_i$	$1\le i\le m-1$	$4(4i-2)$
	$B_i$	$1\le i\le n-m+1$	$4(m-1)+2$
	$C_i$	$1\le i\le m-1$	$2(2i-1)$
	$Y_i$	$1\le i\le n$	2*m*
	$Z_i$	$1\le i\le 2n-1$	2*m*

### Theorem 8.1

*Let* *G* *denote the graph of 2D-lattice of* $TiO_{2}$ *nanotubes*. *The Mostar index of* *G* *is given as follows*:

$$\begin{aligned} Mo(G) = {\left\{ \begin{array}{ll} -24n^4+\frac{1}{3}(144m-188)n^3+\frac{1}{3}(36m^2+132m\\ -108)n^2+\frac{1}{3}(24m^2-36m-4)n-4m^2+4m; &{} m>n \\ 60n^4+\frac{1}{3}(-360m+272)n^3+\frac{1}{3}(36m^2-84m\\ +24)n^2+\frac{1}{3}(312m^2-276m-68)n-48m^3\\ +80m^2-28m; &{} m\le n \end{array}\right. } \end{aligned}$$

**Table 6:** The values of $n_u$ and $n_v$ with respect to the cuts presented in Table 5.

When $m > n$		
type	$n_u$	$n_v$
$A_i$	$6i^2 + 2i$	$-6i^2+12mn-2i-2m$
$B_i$	$12in + 6n^2 -2i + 2n$	$-12in +12mn-6n^2+2i$
		$$-2m-2n$$
$C_i$	$-6i^2+12mn+4i-2m$	$6i^2-4i$
$Y_i$	$2m(3i-2)$	$-6im+12mn+2m$
$Z_i$	$6im-2m+1$	$-6im+12mn-1$

When $m \le n$		
type	$n_u$	$n_v$
$A_i$	$6i^2 + 2i$	$-6i^2+12mn-2i-2m$
$B_i$	$12im + 6m^2 - 10m$	$-12im -6m^2+12mn+8m$
$C_i$	$-6i^2+12mn+4i-2m$	$6i^2-4i$
$Y_i$	$2m(3i-2)$	$-6im+12mn+2m$
$Z_i$	$6im-2m+1$	$-6im+12mn-1$

### Proof

By using the Tables 5 and 6 to obtain the desire results.

$$\begin{aligned} Mo(G) = \sum \limits _{e=uv \in E(G)} |n_u(e)-n_v(e)| \end{aligned}$$

Case-I:When $m>n$, we have

$$\begin{aligned}= & {} \sum _{i=1}^{n} |A_i| |(6i^2 + 2i) - (-6i^2+12mn-2i-2m)|+ \sum _{i=1}^{m-n-1} |B_i| \\{} & {} |(12in + 6n^2 -2i + 2n) - (-12in+12mn-6n^2+2i-2m-2n)|\\{} & {} + \sum _{i=1}^{n} |C_i| |(-6i^2+12mn+4i-2m) - (6i^2-4i)|\\{} & {} + \sum _{i=1}^{n} |Y_i| \times |2m(3i-2) - (-6im+12mn+2m)|\\{} & {} + \sum _{i=1}^{2n-1} |Z_i| \times |(6im-2m+1) - (-6im+12mn-1)|\\= & {} \sum _{i=1}^{n} 4i |(12i^2 + 4i -12mn+2m)|+ \sum _{i=1}^{m-n-1} 4n |(24in+ 12n^2 -4i \\{} & {} +4n -12mn +2m)| + \sum _{i=1}^{n} 2(2i-1) \times |(-12i^2+8i+12mn-2m )|\\{} & {} + \sum _{i=1}^{n} 2m\times |(12im-6m-12mn)|+ \sum _{i=1}^{2n-1} 2m \times |(12im-12mn-2m+2)|\\= & {} \sum _{i=1}^{n} 4i (-12i^2 - 4i + 12mn - 2m)+ \sum _{i=1}^{m-n-1} 4n \times (24in + 12n^2 -4i + 4n \\{} & {} -12mn +2m)+ \sum _{i=1}^{n} 2(2i-1) \times (-12i^2 +8i+12mn-2m )+\sum _{i=1}^{n} 2m \\{} & {} (-12im+6m+12mn)+ \sum _{i=1}^{2n-1} 2m \times (-12im+12mn+2m-2)\\= & {} -24n^4+\frac{1}{3}(144m-188)n^3+\frac{1}{3}(36m^2+132m-108)n^2\\{} & {} +\frac{1}{3}(24m^2-36m-4)n-4m^2+4m \end{aligned}$$

Case-II: Similarly, when $m\le n$, we have

$$\begin{aligned}= & {} \sum _{i=1}^{n} |A_i| |(6i^2 + 2i) - (-6i^2+12mn-2i-2m)| + \sum _{i=1}^{m-n-1} |B_i| \\{} & {} |(12im + 6m^2 -10m) - (-12im+12mn-6m^2+8m)|\\{} & {} + \sum _{i=1}^{n} |C_i| |(-6i^2 +12mn+4i-2m) - (6i^2-4i)| + \sum _{i=1}^{n} |Y_i| \\{} & {} \times |2m(3i-2) - (-6im+12mn+2m)|+ \sum _{i=1}^{2n-1} |Z_i| \\{} & {} \times |(6im-2m+1) - (-6im+12mn-1)|\\= & {} \sum _{i=1}^{n} 4(4i-2) |(12i^2 + 4i -12mn+2m|+ \sum _{i=1}^{m-n-1} 4(m-1)+2 \\{} & {} |(24im + 12m^2 - 18m-12mn)| + \sum _{i=1}^{n} 2(2i-1) \\{} & {} \times |(-12i^2+8i+12mn-2m )|+ \sum _{i=1}^{n} 2m\times |(12im -6m-12mn)|\\{} & {} + \sum _{i=1}^{2n-1} 2m \times |(12im-12mn-2m+2)|\\ \end{aligned}$$

$$\begin{aligned}= & {} \sum _{i=1}^{n} 16i-8 \times (12i^2 + 4i -12mn+2m)+ \sum _{i=1}^{m-n-1} 4m-2 \\{} & {} \times (24im+ 12m^2 - 18m-12mn) + \sum _{i=1}^{n} 4i-2 \times (12i^2\\{} & {} -8i-12mn+2m )+ \sum _{i=1}^{n} 2m\times (-12im +6m+12mn) \\{} & {} + \sum _{i=1}^{2n-1} 2m \times (-12im+12mn+2m-2) \\= & {} 60n^4+\frac{1}{3}(-360m+272)n^3+\frac{1}{3}(36m^2-84m+24)n^2\\{} & {} +\frac{1}{3}(312m^2-276m-68)n-48m^3+80m^2-28m. \end{aligned}$$

$$\square$$

Similarly, the edge Mostar index is calculated in the following theorem for the graphs of titania nanotubes.

### Theorem 8.2

*Let* *G* *denote the graph of 2D-lattice of* $TiO_{2}$ *nanotubes*. *The Mostar index of* *G* *is given as follows*:

$$\begin{aligned} Mo_e(G) = {\left\{ \begin{array}{ll} -40n^4+\frac{1}{3}(240m-256)n^3+\frac{1}{3}(60m^2+324m\\ -174)n^2+\frac{1}{3}(-72m^2+48m-2)n-4m^2+4m; &{} m>n \\ 160m^4+100n^4-(120m-96)n^3-(-180m^2\\ +108m+90)n^2-(320m^3-512m^2+252m38)n\\ -392m^3+328m^2-108m+12.; &{} m\le n \end{array}\right. } \end{aligned}$$

**Table 7:** The values of $m_u$ and $m_v$ with respect to the cuts presented in Table 5.

When $m > n$		
Type	$m_u$	$m_v$
$A_i$	$10i^2-i$	$-10i^2+20mn-3i-4m-4n$
$B_i$	$20in + 10n^2-4i-n$	$-20in +20mn-10n^2+4i$
		$$-4m-7n$$
$C_i$	$-10i^2+20mn+7i$	$10i^2-11i+2-4m-4n$
$Y_i$	$10im-2i-8m+1$	$-10im+20mn+2i+2m-4n-1$
$Z_i$	$10im-2i-4m+1$	$-10im+20mn+2i-2m-4n-1$

When $m \le n$		
Type	$m_u$	$m_v$
$A_i$	$10i^2-i$	$-10i^2+20mn-3i-4m-4n$
$B_i$	$20im + 10m^2 -4i$	$-20im -10m^2+20mn+4i+13m$
	$- 21m +4$	$-4n-2$
$C_i$	$-10i^2+20mn+7i$	$10i^2-11i+2$
	$$-4m-4n$$	
$Y_i$	$10im-2i-8m+1$	$-10im+20mn+2i+2m-4n-1$
$Z_i$	$10im-2i-4m+1$	$-10im+20mn+2i-2m-4n-1$

### Proof

By using Tables 5 and 7 to obtain the results.

$$\begin{aligned} Mo_e(G) = \sum \limits _{e=uv \in E(G)} |m_u(e)-m_v(e)| \end{aligned}$$

Case-I:When $m>n$, we have

$$\begin{aligned}= & {} \sum _{i=1}^{n} |A_i| |(10i^2 -i) - (-10i^2+20mn-3i-4m-4n)| + \sum _{i=1}^{m-n-1} |B_i| \\{} & {} |(20in + 10n^2 -4i - n) - (-20in+20mn-10n^2+4i-4m-7n)|+ \sum _{i=1}^{n} |C_i| \\{} & {} |(-10i^2+20mn+7i)- (10i^2-11i+2-4m-4n)| + \sum _{i=1}^{n} |Y_i| \\{} & {} \times |(10im-2i-8m+1)-(-10im+20mn+2i+2m-4n-1)| +\sum _{i=1}^{2n-1} |Z_i| \\{} & {} \times |(10im-2i-4m+1) - (-10im+20mn+2i-2m-4n-1)| \\= & {} \sum _{i=1}^{n} 4i |(20i^2 + 2i -20mn+4m+4n)|+ \sum _{i=1}^{m-n-1} 4n |(40in+ 20n^2 -8i\\{} & {} + 6n -20mn -4m)| + \sum _{i=1}^{n} 2(2i-1) \times |(-20i^2+18i+20mn\\{} & {} +4m+4n-2 )|+ \sum _{i=1}^{n} 2m\times |(20im -10m-20mn-4i+4n+2)|\\{} & {} + \sum _{i=1}^{2n-1} 2m \times |(20im-4i-20mn-2m+4n+2|\\= & {} \sum _{i=1}^{n} 4i (-20i^2 - 2i +20mn-4m-4n)|+ \sum _{i=1}^{m-n-1} 4n (40in + 20n^2 -8i + 6n \\{} & {} -20mn -4m) + \sum _{i=1}^{n} 2(2i-1) \times (-20i^2+18i+20mn+4m+4n-2 )\\{} & {} + \sum _{i=1}^{n} 2m\times (-20im +10m+20mn+4i-4n-2)+ \sum _{i=1}^{2n-1} 2m \times (-20im\\{} & {} +4i+20mn+2m-4n-2\\= & {} -40n^4+\frac{1}{3}(240m-256)n^3+\frac{1}{3}(60m^2+324m-174)n^2\\{} & {} +\frac{1}{3}(-72m^2+48m-2)n-4m^2+4m. \end{aligned}$$

Case-II: when $m\le n$, we have

$$\begin{aligned} =&\sum _{i=1}^{n} |A_i| |(10i^2 -i) - (-10i^2+20mn-3i-4m-4n)| +\sum _{i=1}^{m-n-1}|B_i|\\&|(20im + 10m^2 -4i - 21m+4) - (-20im +20mn-10m^2+4i\\&-13m-4n-2)|+ \sum _{i=1}^{n} |C_i| |(-10i^2+20mn+7i-4m-4n) \\&- (10i^2-11i+2)| + \sum _{i=1}^{n} |Y_i| \times |(10im-2i-8m+1) - (-10im\\&+20mn+2i+2m -4n-1)|+ \sum _{i=1}^{2n-1} |Z_i| \times |(10im-2i-4m\\&+1)-(-10im+20mn+2i-2m-4n-1)|\\ =&\sum _{i=1}^{n} 4(4i-2) |(20i^2 + 2i -20mn+4m+4n)|+\sum _{i=1}^{m-n-1} (4(m-1)+2)\\&|(40im + 20m^2 -8i + 4n-20mn-34m+6)|+\sum _{i=1}^{n} 2(2i-1) \\&\times |(-20i^2+18i+20mn-4m-4n-2 )|+\sum _{i=1}^{n} 2m\times |(20im -10m\\&-20mn-4i+4n+2)|+\sum _{i=1}^{2n-1} 2m \times |(20im-4i-20mn-2m\\&-4n+2)|\\ =&\sum _{i=1}^{n} 4i-1 \times (20i^2 + 2i -20mn+4m+4n) +\sum _{i=1}^{m-n-1} 4m-2 \\&\times (40im+ 20m^2 -8i + 4n -20mn -34m+6) + \sum _{i=1}^{n} 2(2i-1) \\&\times (20i^2-18i-20mn+4m+4n+2)+ \sum _{i=1}^{n} 2m\times (-20im+10m\\&+20mn+4i-4n-2)+\sum _{i=1}^{2n-1} 2m \times (-20im+4i+20mn+2m\\&+4n-2) =160m^4+100n^4-(120m-96)n^3-(-180m^2+108m+90)n^2\\&-(320m^3-512m^2+252m+38)n-392m^3+328m^2-108m+12. \end{aligned}$$

$$\square$$

## Concluding remarks and discussion

A topological index is a molecular graph invariant which correlates the physico-chemical properties of a molecular graph with a number. This paper deals with some degree-based topological indices of an infinite class of Titania nanotubes $TiO_2[m,n]$. The output of all these geometrical indices of nanotubes is of practical interest. For instance, the design of supports possessing certain properties for controlled drug release. Here we represented the numerical comparison of different indices for $TiO_2[m,n]$ for different values of *n* and *m*.

**Table:** 

When $m=1 \le n$					
$TiO_2(n,1)$	*F*(*G*)	${\overline{F}}(G)$	${\overline{M}}(G)$	*Mo*(*G*)	$M o_e(G)$
n=1	192	384	132	16	-40
n=2	512	2848	812	676	1140
n=3	832	7616	2068	3968	6996
n=4	1152	14688	3900	13316	23384

When $m=5>n$					
$TiO_2$	*F*(*G*)	${\overline{F}}(G)$	${\overline{M}}(G)$	*Mo*(*G*)	$M o_e(G)$
n=1	1904	28528	6672	696	656
n=2	4784	122464	32416	3168	4684
n=3	7664	336080	77360	7536	12452
n=4	10544	616576	141504	14384	25048

In this paper, we studied the a recently defined topological index known as the forgotten index or *F*-index of our class of titania nanotubes. We also calculated two co-type indices of our class of nanotubes namely F-coindex and the first version of the Zagreb coindex.

**Figure 8:**
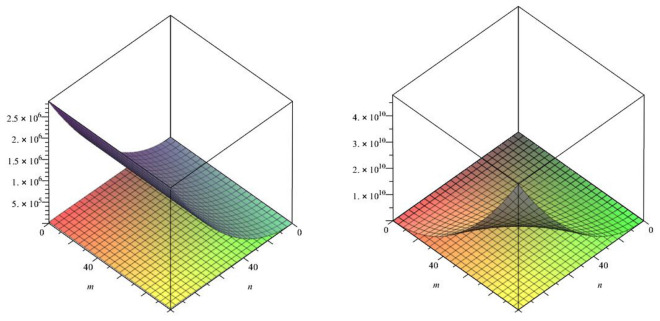
The lattices of $TiO_{2}[m,n]$ for $m=1$ and $n=1,2,3$.

In above mention graphical representation in Fig. 8, we have graphical comparison of the *F*-index for m = 1 and m > 1 with their *F*-coindex which clearly shows that for increasing order, *F* index and *F*-coindex are also increasing in projectile way with different angle of elevation.

**Figure 9:**
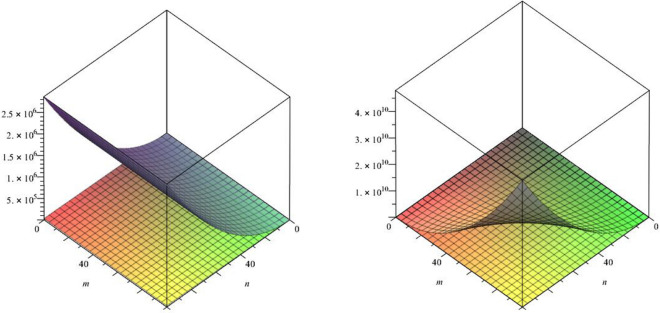
The lattices of $TiO_{2}[m,n]$ for $m=1$ and $n=1,2,3$.

In above mention graphical representation in Fig. 9, we have graphical comparison of the *F*-index with the Zagreb index of first kind for m = 1 and for m > 1 which clearly shows that for increasing order, respective indices are also increasing in distinct angle.

**Figure 10:**
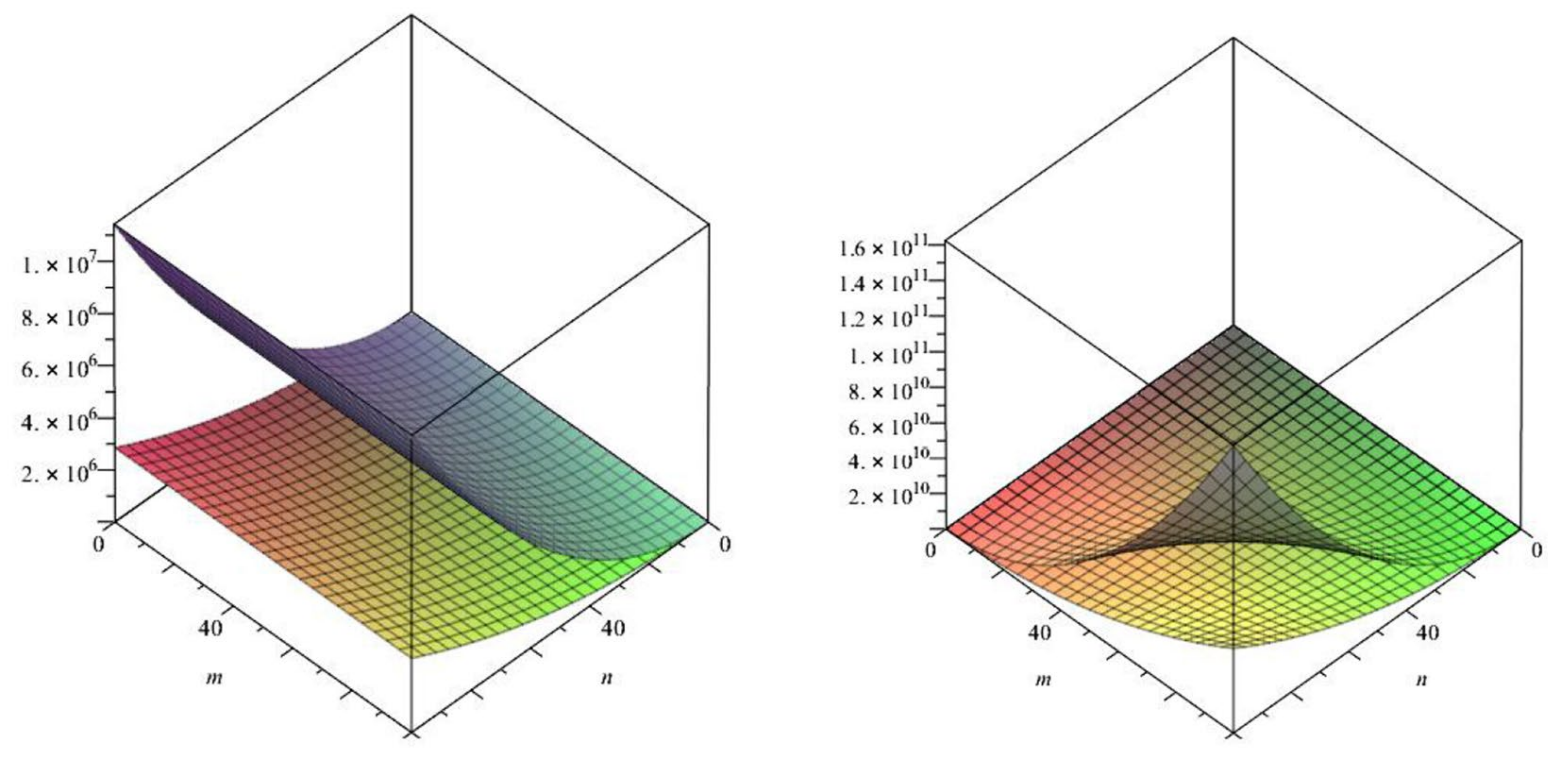
The lattices of $TiO_{2}[m,n]$ for $m=1$ and $n=1,2,3$.

In above mention graphical representation in Fig. 10, we have graphical comparison of the Co-index of *F* and Zagreb index of first kind for m = 1 and for m > 1 which clearly shows that both indices are increasing with their respective different angles.

**Figure 11:**
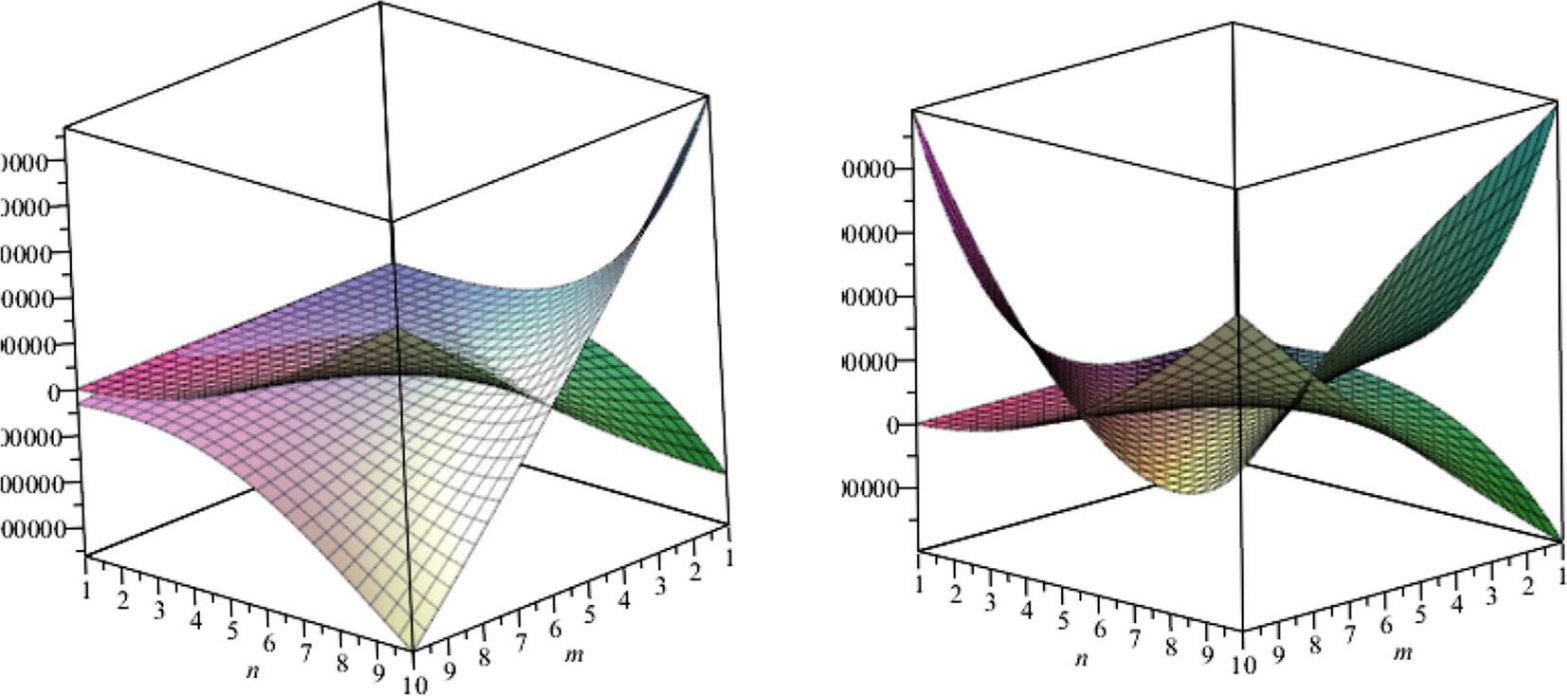
The lattices of $TiO_{2}[m,n]$ for different values of*m* and *n*.

In above mention graphical representation in Fig. 11, we have graphical comparison of Mostar index for vertex and edge when $m>n$ and $m \le n$ which clearly show that both indices are monotonically increasing with their respective different angles.

## Data Availability

All data generated or analysed during this study are included in this published article. The data used and analysed during the current study available from the corresponding author on reasonable request.

## References

[1] Hosoya H (1971). Topological index: A newly proposed quantity characterizing the topological nature of structural isomers of saturated hydrocarbons. Bull. Chem. Soc. Jpn..

[2] Gutman I, Trinajstic N (1972). “Graph theory and molecular orbitals. Total $$\pi$$-electron energy of alternant hydrocarbons. Chem. Phys. Lett..

[3] Gutman I, Ručič B, Trinajstic N, Wilcox CF (1975). “Graph theory and molecular orbitals. XII”, Acyclic Polyenes. J. Chem. Phys..

[4] Furtula B, Gutman I (2015). A forgotten topological index. J. Math. Chem..

[5] De, N., Nayeem, A. & Pal, A. *The F-Coindex of Some Graph Operations*, vol. 5 (Springer Plus, 2016). 10.1186/s40064-016-1864-7 PMC4771675 27026915

[6] Doslic T (2008). Vertex weighted Wiener polynomials for composite graphs. Ars Math. Contemp..

[7] Dolić T, Martinjak I, Krekovski R, Spuević ST, Zubac I (2018). Mostar index. J. Math. Chem..

[8] Havare OC (2022). Some inequalities on the Mostar index: CREAT. Math. Inform..

[9] Tratnik N (2021). Computing the Mostar index in networks with applications to molecular graphs. Iran. J. Math. Chem..

[10] Akhter S, Iqbal Z, Aslam A, Gao W (2021). Computation of Mostar index for some graph operations. Int. J. Quantum Chem..

[11] Došlić T, Martinjak I, Škrekovski R (2018). Mostar index. J. Math. Chem..

[12] Ali A, Došlić T (2021). Mostar index: Results and perspectives. Appl. Math. Comput..

[13] Ghorbani M, Rahmani S, Eslampoor M (2020). Some new results on Mostar index of graphs. Iran. J. Math. Chem..

[14] Ashrafi AR, Doslic T, Hamzeh A (2010). The Zagreb coindices of graph operations. Discr. Appl. Math..

[15] Ashrafi AR, Doslic T, Hamzeh A (2011). Extremal graphs with respect to the Zagreb coindices. MATCH Commun. Math. Comput. Chem..

[16] HosseinZadeh S, Hamzeh A, Ashrafi AR (2010). Extermal properties of Zagreb coindices and degree distance of graphs. Miskolc Math. Notes.

[17] Hua H, Ashrafi A, Zhang L (2012). More on Zagreb coindices of graphs. Filomat.

[18] Wang M, Hua H (2012). More on Zagreb coindices of composite graphs. Int. Math. Forum.

[19] Das KC, Gutman I, Horoldagva B (2012). Comparing Zagreb indices and coindices of trees. MATCH Commun. Math. Comput. Chem..

[20] Doslic T, Furtula B, Graovac A, Gutman I, Moradi S, Yarahmadi Z (2011). On vertex degree based molecular structure descriptors. MATCH Commun. Math. Comput Chem..

[21] Vukicevic ZK, Popivorda G (2014). Chemical trees with extreme values of Zagreb indices and coindices. Iran. J. Math. Chem..

[22] Malik MA (2018). Two degree-distance based topological descriptors of some product graphs. Discret. Appl. Math..

[23] Baca M, Horváthová J, Mokriŝová M, Suhányiová A (2015). On topological indices of fullerenes App. Math. Comput..

[24] Ghorbani M (2014). Some new results on the nullity of moecular graphs. Studia Ubb Chem..

[25] Havare OC, Havare AK (2020). Computation of the forgotten topological index and co-index for carbon base nanomaterial. Polycycl. Aromat. Compd..

[26] Ashrafi AR, Nikzad P, Austin K (2009). Digest J. Nanomater. Biostruct..

[27] Imran M, Malik MA, Javed R (2021). On Szeged-type indices of titanium oxide TiO2 nanotubes. Int. J. Quantum Chem..

[28] Klavzar S (2008). A Bird’s eye view of the cut-method and a survey of its applications in chemical graph theory. MATCH Commun. Math. Comput. Chem..

[29] Evarestov RA, Zhukovskii YF, Bandura AV, Piskunov S (2011). Symmetry and models of single-walled $$TiO_2$$ nanotubes with rectangular morphology. Cent. Eur. J. Phys..

[30] Wikipedia public domain. https://commons.wikimedia.org/wiki/File:Anatase-unit-cell-3D-balls.png.

[31] Mayura Draw. http://www.mayura.com/.

[32] Sevda J, Baharak M, Hadi H, Sajjad J, Gholikhani T, Tayebi L (2020). Biomedical applications of $$TiO_2$$ nanostructures recent advances. Int. J. Nanomed..

[33] Adobe Illustrator CC (2019). https://creative.adobe.com/products/download/illustrator.

[34] Irshad MA, RabNawazb MZ, Ur Rehman M, Adrees M, Rizwana S, Ali S, Ahmad S, Tasleem S (2021). Synthesis, characterization and advanced sustainable applications of titanium dioxide nanoparticles: A review, synthesis, characterization and advanced sustainable applications of titanium dioxide nanoparticles: A review. Ecotoxicol. Environ. Saf..

[35] Nabi I, Bacha A-U-R, Ahmad F, Zhang L (2021). Application of titanium dioxide for the photocatalytic degradation of macro and micro-plastics: A review. J. Environ. Chem. Eng..

